# Prediction and management of strangulated bowel obstruction: a multi-dimensional model analysis

**DOI:** 10.1186/s12876-022-02363-1

**Published:** 2022-06-22

**Authors:** Wei-xuan Xu, Qi-hong Zhong, Yong Cai, Can-hong Zhan, Shuai Chen, Hui Wang, Lin Lin, Ying-qian Geng, Ping Hou, Xian-qiang Chen, Jun-rong Zhang

**Affiliations:** 1 Fujian Medical University, No.1 Xuefu bei Road, Fuzhou, 350122 Fujian Province China; 2 Immunotherapy Institute, Fujian Medical University, No.1 Xuefu bei Road, Fuzhou, 350122 Fujian Province China; 3 Department of General Surgery (Emergency Surgery), Fujian Medical University Union Hospital, No.29 Xinquan Road, Fuzhou, 350001 Fujian Province China; 4 Department of Radiology, Fujian Medical University Union Hospital, No.29 Xinquan Road, Fuzhou, 350001 Fujian Province China

**Keywords:** Strangulated bowel obstruction, Radiological, Multi-dimensional model, Risk factors, Indications

## Abstract

**Background:**

Distinguishing strangulated bowel obstruction (StBO) from simple bowel obstruction (SiBO) still poses a challenge for emergency surgeons. We aimed to construct a predictive model that could distinctly discriminate StBO from SiBO based on the degree of bowel ischemia.

**Methods:**

The patients diagnosed with intestinal obstruction were enrolled and divided into SiBO group and StBO group. Binary logistic regression was applied to identify independent risk factors, and then predictive models based on radiological and multi-dimensional models were constructed. Receiver operating characteristic (ROC) curves and the area under the curve (AUC) were calculated to assess the accuracy of the predicted models. Via stratification analysis, we validated the multi-dimensional model in the prediction of transmural necrosis both in the training set and validation set.

**Results:**

Of the 281 patients with SBO, 45 (16.0%) were found to have StBO, while 236(84.0%) with SiBO. The AUC of the radiological model was 0.706 (95%CI, 0.617–0.795). In the multivariate analysis, seven risk factors including pain duration ≤ 3 days (OR = 3.775), rebound tenderness (OR = 5.201), low-to-absent bowel sounds (OR = 5.006), low levels of potassium (OR = 3.696) and sodium (OR = 3.753), high levels of BUN (OR = 4.349), high radiological score (OR = 11.264) were identified. The AUC of the multi-dimensional model was 0.857(95%CI, 0.793–0.920). In the stratification analysis, the proportion of patients with transmural necrosis was significantly greater in the high-risk group (24%) than in the medium-risk group (3%). No transmural necrosis was found in the low-risk group. The AUC of the validation set was 0.910 (95%CI, 0.843–0.976). None of patients in the low-risk and medium-risk score group suffered with StBO. However, all patients with bowel ischemia (12%) and necrosis (24%) were resorted into high-risk score group.

**Conclusion:**

The novel multi-dimensional model offers a useful tool for predicting StBO. Clinical management could be performed according to the multivariate score.

**Supplementary Information:**

The online version contains supplementary material available at 10.1186/s12876-022-02363-1.

## Background

Small bowel obstruction (SBO) is a common disease, accounting for 12%-16% of all surgical admissions in the United States[1]. SBO can be divided into simple bowel obstruction (SiBO) and strangulated bowel obstruction (StBO). SiBO is usually resolved by nonoperative management, including bowel rest, nasogastric tubes and tube decompression, reducing the risk of emergency surgery[2]. Conversely, StBO requires immediate surgical intervention[3], as StBO may result in severe complications, including bowel perforation, peritonitis and septic shock, which increase the mortality of SBO up to 25%[4, 5]. In the case of bowel transmural necrosis, the mortality dramatically increases to 50%[6]. However, only 1/3 of StBO patients have the classical traits of abdominal pain, hematochezia and fever, and the remaining patients have nonspecific symptoms such as diarrhea, vomiting and bloating[7]. Consequently, it is difficult to accurately diagnose and intervene in StBO in the early stage. How to distinguish StBO from SiBO still poses a challenge to emergency surgeons.

Traditionally, clinical findings serve as major models for the prediction of StBO[8–10]; however, the accuracy of these models remains unsatisfactory[11]. More focus has been placed on radiological characteristics[12–14], whereas the diagnostic performance of CT revealed poor prospective prediction[8, 15]. CTA (computed tomography angiography) is the gold standard of predicting bowel ischemia with 83–100% sensitivity and 61–93% specificity[16]. However, CTA is rarely performed in emergency situations due to its high cost, insufficient medical support, potential allergic reaction and high risk of nephropathy induced by iodine[17–19]. In previous studies for the detection of laboratory biomarkers to evaluate StBO, only L-lactate was deemed an effective biomarker for the prediction of bowel ischemia, with 78% sensitivity and 48% specificity[6, 20]. Therefore, predictive models integrating clinical features, laboratory tests and radiological characteristics need to be studied for the prediction of StBO.

To date, few studies have focused on the prediction of bowel transmural necrosis for SBO, most of which enrolled patients with acute mesenteric ischemia (AMI)[21–23]. Among these studies, only laboratory biomarkers were primarily considered indicative factors of bowel transmural necrosis[21, 22]. To our knowledge, no efforts have been made to predict transmural necrosis in patients with SBO. Therefore, a multi-dimensional model to predict transmural necrosis in patients with SBO is urgently needed.

In this study, we constructed an accurate predictive model consisting of clinical features, laboratory tests and radiological characteristics for the diagnosis of StBO. Based on the predictive model, we could distinctly discriminate StBO from SiBO, especially for transmural necrosis from simple bowel ischemia.

## Methods

### Patient population

**Fig. 1:**
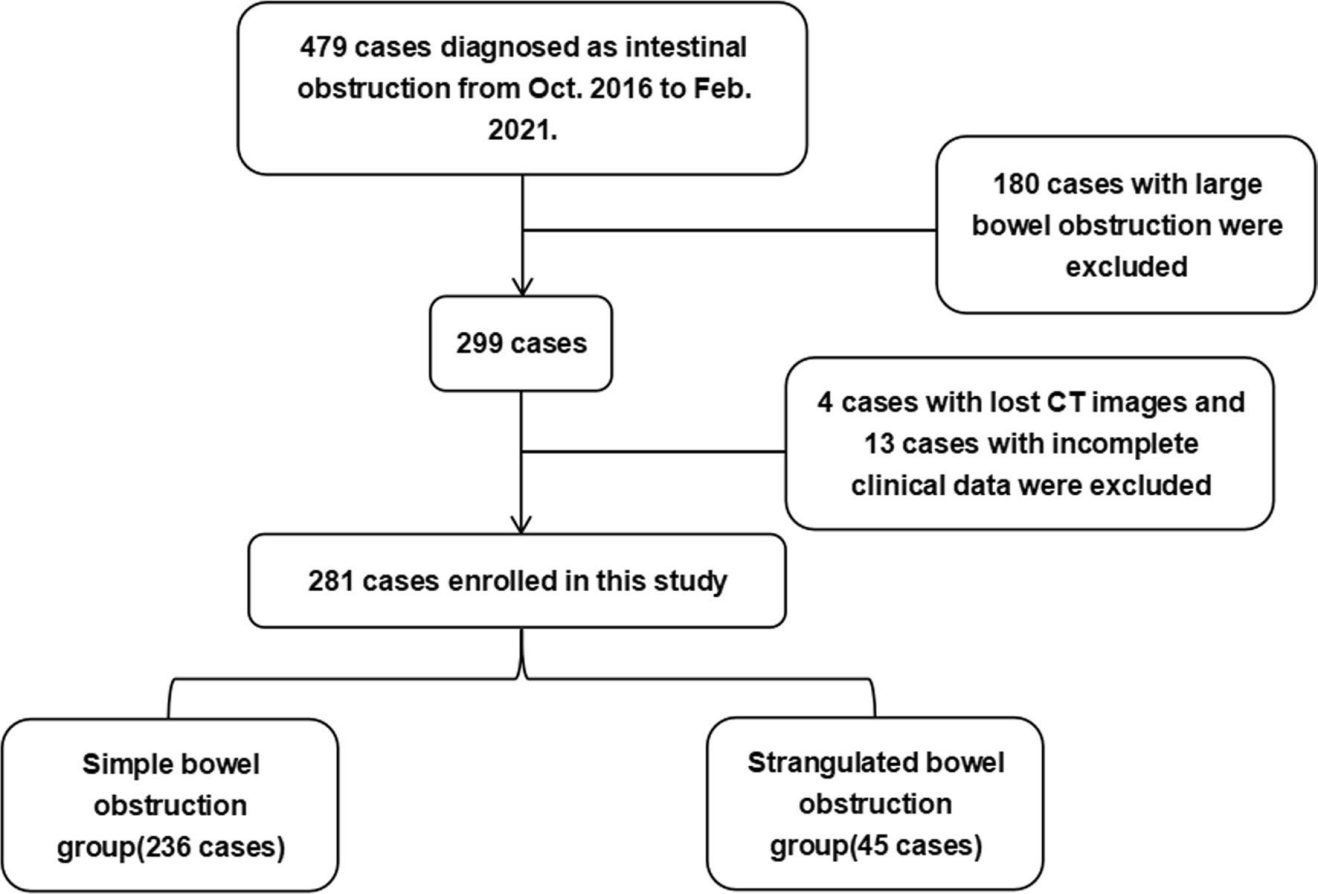
Workflow of this study

We divided patients in our cohort into two group including training set and validation set. In the training set, from October 2016 to February 2021, 479 patients diagnosed with intestinal obstruction at Fujian Medical University Union Hospital were included in the retrospective study. After excluding 180 patients with large bowel obstruction, 4 patients with missing CT images and 13 patients with incomplete clinical data, 281 patients were recruited in the final study (shown in Fig. 1). For the validation set, 80 patients diagnosed as intestinal obstruction accepted treatment in our unit from March 2021 to Mar 2022.

The patients were divided into two groups according to the pathological confirmation of intestinal ischemia: a simple bowel obstruction (SiBO) group and a strangulated bowel obstruction (StBO) group. The study protocol was approved by the Institutional Review Board of Fujian Medical University Union Hospital (FJMUUH), and all patients provided written informed consent for the procedure.

### Clinical characteristics and laboratory tests

Clinical parameters, including pain duration, symptoms of abdominal pain, tenderness, rebound tenderness, and bowel sounds, and laboratory tests, including white blood cell count (WBC), prothrombin time (PT), and potassium, sodium, blood urea nitrogen (BUN), and D-dimer (DDI) levels, were recorded in our database for intestinal bowel obstruction. Categorical variables, especially potassium, sodium, BUN, PT and DDI levels, were transformed from continuous variables according to laboratory references[24–28]. The levels of WBC and NE%, were sorted by quartile. In addition, procalcitonin (PCT) levels were divided into three categories based on a previous study[20].

### CT findings

**Fig. 2:**
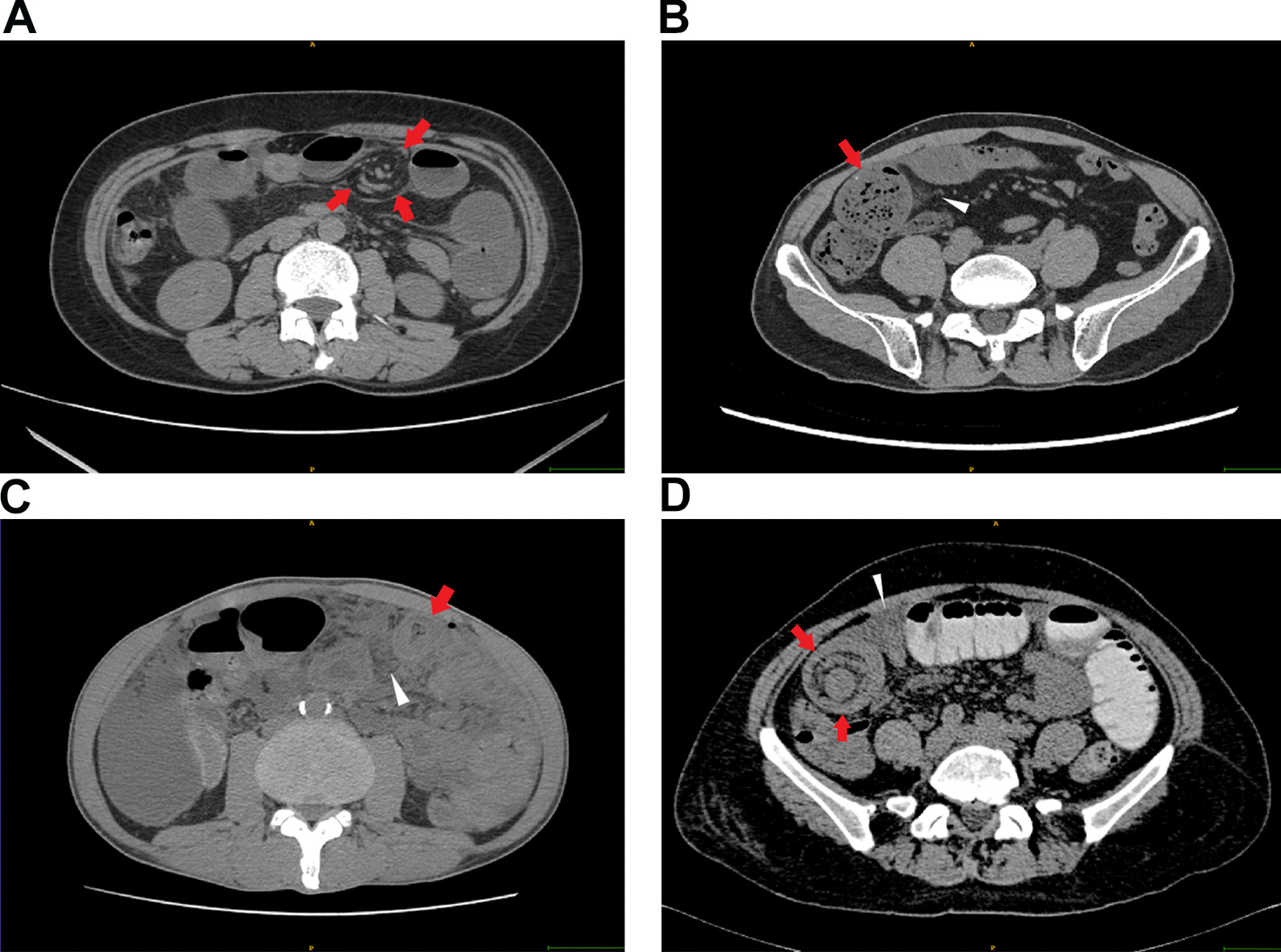
Images of CT findings. **A** a 49-year-old woman with adhesive small bowel obstruction. Axial CT of the abdomen confirmed the spiral sign of small bowel (red arrow). **B** a 51-year-old man with adhesive small bowel obstruction. Axial CT of the pelvis confirmed the small bowel feces sign(red arrow) and the mesenteric fluid (white triangle). **C** a 51-year-old man with inflammatory small bowel obstruction. Dilated, thickened loops of small bowel (red arrow) and mesenteric fluid (white triangle) could be observed. **D** a 70-year-old woman with intussusception. Axial CT images showed the concentric circle sign of small bowel (red arrow) and ascites (white triangle)

All patients with suspected SBO underwent CT scans before receiving treatment. The features of the CT scans recorded in this study were separated into mesenteric fluid, ascites, spiral signs, concentric circle signs, small bowel feces signs, and edema of the bowel wall[4, 29–33]. All CT scan images were cross-reviewed and judged by two senior radiologists (radiologist Lin Lin had 10 years of experience in abdominal radiology, and radiologist Ying-qian Geng had 8 years of experience in general radiology). The discriminate portions were independently judged by a general surgeon, Xian-qiang Chen who had over 10 years of experience in abdominal emergency surgery. The definitions of CT characteristics were showed in Fig. 2 and supplied in Additional file 1: Table S2.

### Statistical analysis

The differences between the two groups were compared using the chi-square test or Fisher’s exact test for categorical variables. For continuous variables, we used an independent t-test. For continuous nonparametric variables, the Wilcoxon rank-sum test was adopted to analyze the differences between the groups. Independent risk factors including pain duration, rebound tenderness, bowel sounds, levels of potassium, sodium, BUN and radiological score, were finally confirmed via binary logistic regression and none of them showed feature correlation via co-linearity analysis. We also extracted a risk score formula based on the seven independent risk factors. RS = [1.328 ×(pain duration level) + 1.649 ×(rebound tenderness level) + 1.611 ×(bowel sounds level) + 1.307 ×(potassium level) + 1.323 ×(sodium level) + 1.470 ×(BUN level) + 2.422 ×(Radiological score) − 6.009]. Receiver operating characteristic (ROC) curves and the area under the curve (AUC) were calculated to assess the accuracy of the predicted models. A logistic nomogram was generated by using tools in Hiplot (https://hiplot.com.cn), a comprehensive web platform for scientific data visualization. The other statistical analyses were performed with SPSS software (SPSS, version 23.0, SPSS Inc.).

## Results

### Background and clinical-laboratory features

Of the 281 patients with SBO who were included in this study, 45 (16.0%) were found to have StBO, while 236 (84.0%) were found to have SiBO. No remarkable differences were observed between the groups for the baseline parameters, including age, sex, BMI, and comorbidity status (all *p* value > 0.05, Table 1). Via univariate analysis, several clinical characteristics, including pain duration (*p* = 0.036), abdominal pain (*p* = 0.018), tenderness (*p* = 0.020), rebound tenderness (*p* < 0.001), and bowel sounds (*p* = 0.014), were significantly different between the two groups. High levels of inflammatory biomarkers, such as WBC (*p* = 0.029) and NE% (*p* = 0.007), and abnormal electrolyte and metabolic changes, such as low levels of sodium (*p* = 0.009), abnormal potassium (*p* = 0.003), and high levels of BUN (*p* < 0.001) and glucose (*p* = 0.002), were closely related to bowel ischemia. In the validation set, rebound tenderness degree (*p* < 0.001), the level of WBC (*p* = 0.029) and NE% (*p* = 0.007) also significantly deteriorated in the StBO group compared with SiBO group (Additional file 1: Table S7).

**Table 1:** Compared the clinical and laboratory characteristics of the patients with or without strangulated bowel obstruction

Characteristics	SiBO (n = 236)	StBO (n = 45)	*p*-value
Gender, n(%)			0.124
male	164(69.5%)	26(57.8%)	
female	72(30.5%)	19(42.2%)	
Age(median)	61	63	0.421*
BMI, n(%)			0.432
18.5–23.9	114(59.1%)	19(52.8%)	
≤ 18.5	45(23.3%)	12(33.3%)	
> 23.9	34(17.6%)	5(13.9%)	
Comorbidity, n(%)			0.406
none	175(74.2%)	36(80.0%)	
yes	61(25.8%)	9(20.0%)	
Pain duration, n(%)			**0.036**
≤ 3 days	133(56.6%)	33(73.3%)	
> 3 days	102(43.4%)	12(26.7%)	
History of abdominal operation, n(%)			0.716
none	62(26.3%)	13(28.9%)	
yes	174(73.7%)	32(71.1%)	
Temperature(median)	36.6	36.6	0.584*
Abdominal pain, n(%)			0.012**
none or mild	35(14.9%)	1(2.2%)	
moderate	142(60.4%)	26(57.8%)	
severe	58(20.7%)	18(40.0%)	
Abdominal distention, n(%)			0.761
none	63(26.7%)	13(28.9%)	
yes	173(73.3%)	32(71.1%)	
Vomiting, n(%)			0.702
none	75(31.8%)	13(28.9%)	
yes	161(68.2%)	32(71.1%)	
Retention of stool and flatus, n(%)			0.077
none	96(40.7%)	12(26.7%)	
yes	140(59.3%)	33(73.3%)	
Tenderness, n(%)			0.038**
none	35(14.8%)	1(2.2%)	
yes	201(85.2%)	44(97.8%)	
Rebound tenderness, n(%)			** < 0.001**
none	190(80.5%)	22(48.9%)	
yes	46(19.5%)	23(51.1%)	
Bowel sounds, n(%)			**0.014**
normal	104(44.1%)	13(28.9%)	
none or low	82(34.7%)	26(57.8%)	
high or hyperactive	50(21.2%)	6(13.3%)	
WBC, (10^9/L), n(%)			**0.029**
≤ 75% quartile	183(77.5%)	28(62.2%)	
> 75% quartile	53(22.5%)	17(37.8%)	
NE%, n(%)			**0.007**
≤ 75% quartile	186(78.8%)	27(60.0%)	
> 75% quartile	50(21.2%)	18(40.0%)	
HCO3-, n(%)(mean)	23.78	23.62	0.789
PCT,n(%)		0.080**	
< 0.02	4(3.4%)	1(6.2%)	
0.02–1	102(87.9%)	11(68.8%)	
> 1	10(8.6%)	4(25%)	
Potassium, n(%)			0.003**
3.5–5.5	204(86.8%)	32(71.1%)	
≤ 3.5	30(12.8%)	10(22.2%)	
> 5.5	1(0.4%)	3(6.7%)	
Sodium, n(%)			**0.009**
> 135	196(83.4%)	30(66.7%)	
≤ 135	39(16.6%)	15(33.3%)	
Glucose(median)	6.80	8.34	0.002*
BUN, n(%)			** < 0.001**
≤ 8.3	185(81.5%)	24(57.1%)	
> 8.3	42(18.5%)	18(42.9%)	
PT, n(%)			0.056**
≤ 16 s	219(94.0%)	38(84.4%)	
> 16 s	14(6.0%)	7(15.6%)	
DDI, n(%)			0.251
≤ 0.5	29(13.6%)	3(7.1%)	
> 0.5	185(86.4%)	39(92.9%)	

*SiBO* simple bowel obstruction, *StBO* strangulated bowel obstruction, *BMI* body mass index, *WBC* white blood cell, *NE%* neutrophil percentage, *PCT*: procalcitonin, *BUN* blood urea nitrogen, *PT* prothrombin time, *DDI* D-dimer

Values marked with “*” were compared using Wilcoxon rank-sum test

Values marked with “**” were adjusted *p*-values

### Univariate and multivariate analyses of radiological characteristics

Through univariate analysis of the radiological characteristics, we determined that StBO was closely related to the presence of mesenteric fluid (*p* = 0.018), ascites (*p* = 0.002), bowel spiral signs (*p* < 0.001) and edema of the bowel wall (*p* = 0.037) (Table 2). Via binary logistic regression analysis, we defined only ascites (OR = 4.067, 95% CI: 1.506–10.983, *p* = 0.006) and bowel spiral signs (OR = 5.506, 95% CI: 2.609–11.623, *p* < 0.001) as independent risk factors for StBO. Similarly, ascites (*p* = 0.003) and bowel spiral signs(*p* = 0.080) also obviously manifested in StBO group in the validation set(Additional file 1: Table S7).

**Table 2:** Univariate and multivariate analysis of CT findings in patients with or without StBO

	Univariate analysis(n = 281)			Multivariate analysis(n = 281)	
CT characteristics	SiBO (n = 236)	StBO (n = 45)	*p*-value	OR (95%*CI*)	*p*-value
Mesenteric fluid			**0.018**		
none	44(18.6%)	2(4.4%)			
yes	192(81.4%)	43(95.6%)			
Ascites			**0.002**	4.067 (1.506–10.983)	**0.006**
none	81(34.3%)	5(11.1%)			
yes	155(65.7%)	40(88.9%)			
Spiral signs			** < 0.001**	5.506 (2.609–11.623)	** < 0.001**
none	211(89.4%)	27(60.0%)			
yes	25(10.6%)	18(40.0%)			
Concentric circle sign			0.476**		
none	225(95.3%)	42(93.3%)			
yes	11(4.7%)	3(6.7%)			
Small bowel feces sign			0.901		
none	113(47.9%)	22(48.3%)			
yes	123(52.1%)	23(51.1%)			
Edema of bowel wall			**0.037**		
none	85(36.0%)	9(20.0%)			
yes	151(64.0%)	36(80.0%)			
Bowel wall thickness (median)	3.33	3.48	0.110*		

The bold indicates that the parameters have statistical difference (*p* < 0.05)

*SiBO* simple bowel obstruction, *StBO* strangulated bowel obstruction, *OR* odds ratio,

*CI* confidence interval

Values marked with “*” were compared using Wilcoxon rank-sum test

Values marked with “**” were adjusted *p*-values

**Fig. 3:**
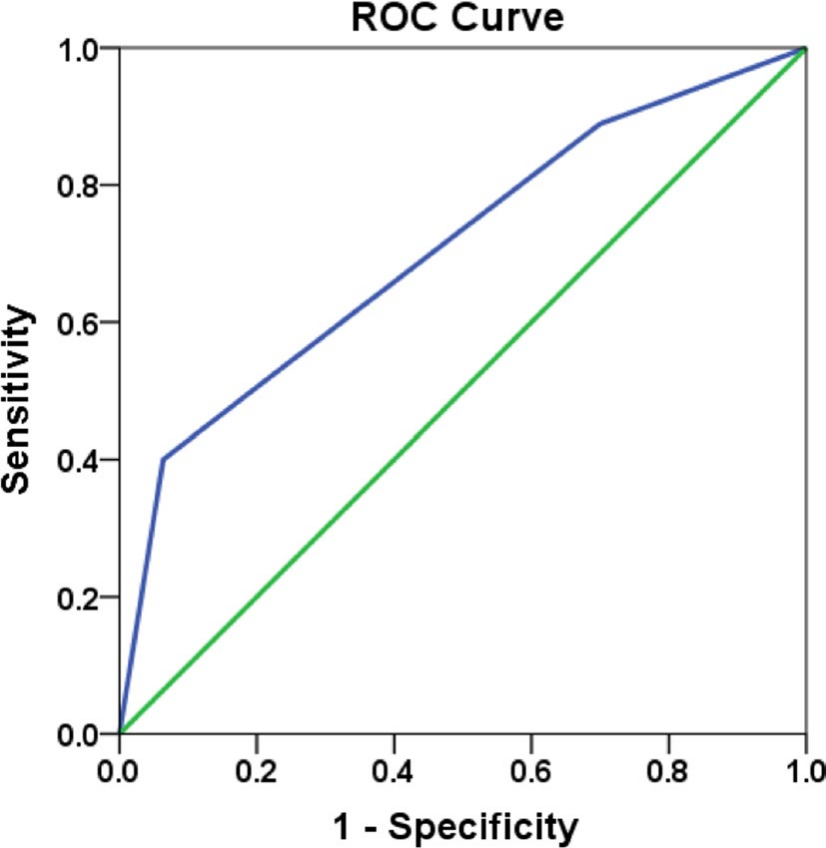
Receiver operating characteristic (ROC) curve for the radiological prediction model. The area under the curve was 0.706(95% CI,0.617–0.795)

Based on the results of multivariate analysis, we built a radiological scoring system to predict the occurrence of StBO. The area under the curve (AUC) of the receiver operating characteristic (ROC) curve for this model was 0.706 (95% CI, 0.617–0.795) (Fig. 3). Furthermore, we observed that the discriminative ability of this model was better when comparing the radiological score of the 2 group with the score of the 0 group (54.5% vs. 6.6%). However, it was difficult to separate the radiological score of the 1 group from the score of the 0 group (12.8% vs. 6.6%) (Additional file 1: Table S3.).

### Multi-dimensional analysis and model construction

**Fig. 4:**
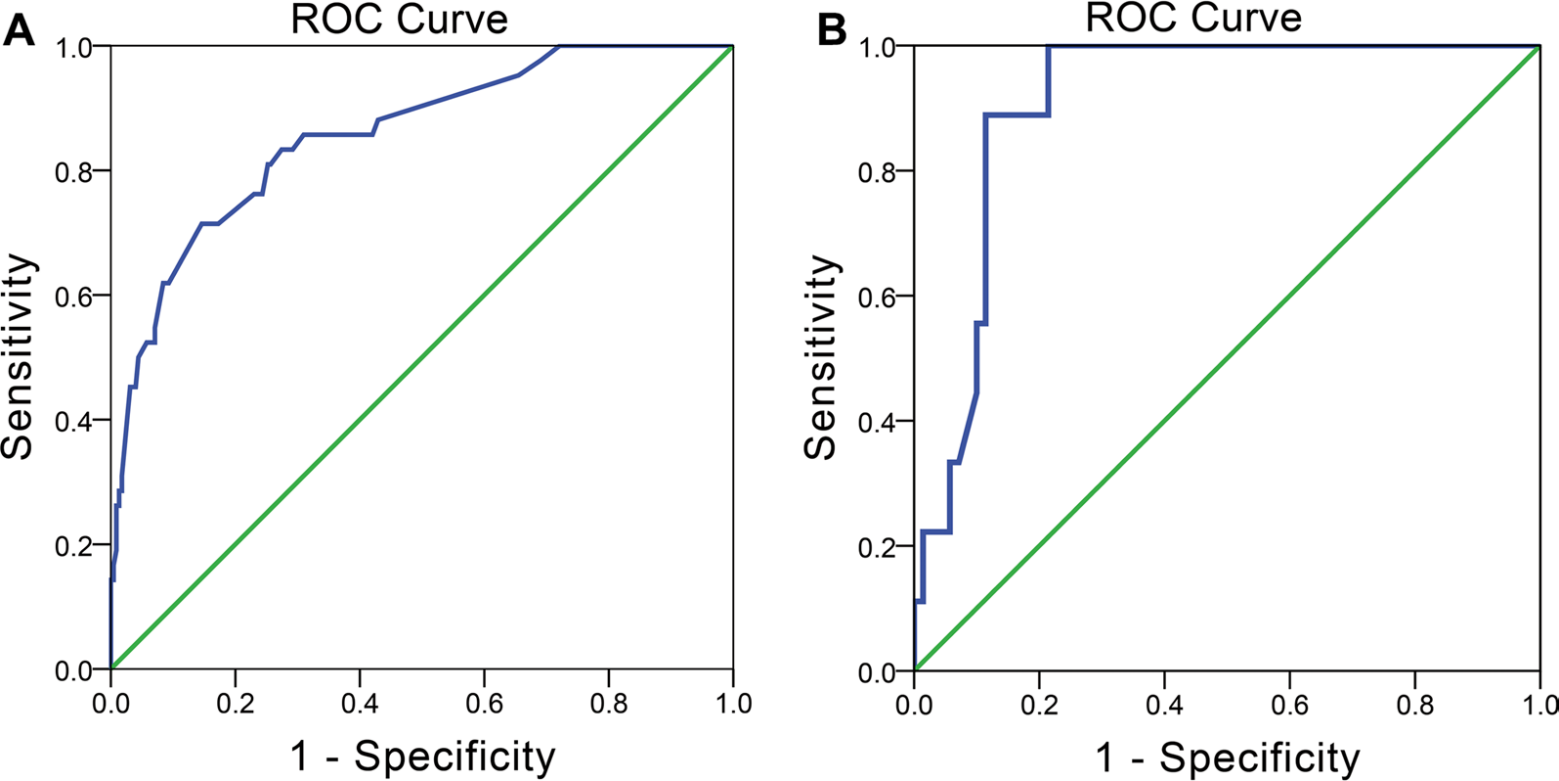
Receiver operating characteristic (ROC) curve for prediction model and external validation set. The areas under the curve were 0.857(95% CI,0.793–0.920), 0.910(95%CI, 0.843–0.976), respectively

**Fig. 5:**
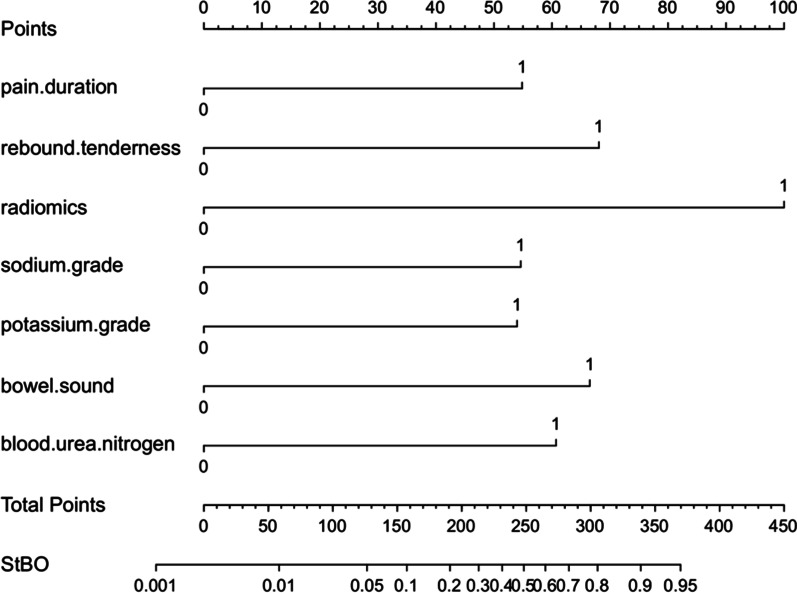
Nomogram for the predictive multi-dimensional model of StBO

Furthermore, we analyzed all essential factors (*p* value < 0.05) from clinical characteristics, laboratory tests and radiological characteristics via binary logistic regression. To obtain better discrimination ability, we transformed three factors, bowel sounds, potassium level and radiological score, into two categories of variables. Finally, we found that pain duration (OR = 3.775), rebound tenderness (OR = 5.201), bowel sounds (OR = 5.006), levels of potassium (OR = 3.696), sodium (OR = 3.753) and BUN (OR = 4.349) and radiological score (OR = 11.264) were independent risk factors for the prediction of StBO (*p* value < 0.05, Table 3). Based on the regression coefficient for each factor, we constructed a multi-dimensional model with an AUC value of 0.857 (95% CI: 0.793–0.920) (Fig. 4. A, model formula is shown in Fig. 7). A nomogram was also drawn to directly calculate the probability of the occurrence of StBO (Fig. 5). As the risk factors accumulated, the incidence of StBO dramatically increased.

**Table 3:** Multi-dimensional analysis for StBO

characteristics	Multivariate analysis		
	Regression coefficient	OR (95%*CI*)	*p*-value
Pain duration(≤ 3 days)	1.328	3.775(1.429–9.973)	**0.007**
Rebound tenderness	1.649	5.201(2.241–12.069)	** < 0.001**
Low-to-absent bowel sounds	1.611	5.006(1.244–20.151)	**0.023**
Low potassium	1.307	3.696(1.184–11.533)	**0.024**
Low sodium	1.323	3.753(1.483–9.498)	**0.005**
High BUN	1.470	4.349(1.793–10.552)	**0.001**
High radiological score	2.422	11.264(4.086–31.047)	** < 0.001**

The bold indicates that the parameters have statistical difference (*p* < 0.05)

*BUN* blood urea nitrogen, *OR* odds ratio, *CI* confidence interval

### Validation of multi-dimensional models for the prediction of StBO

**Fig. 6:**
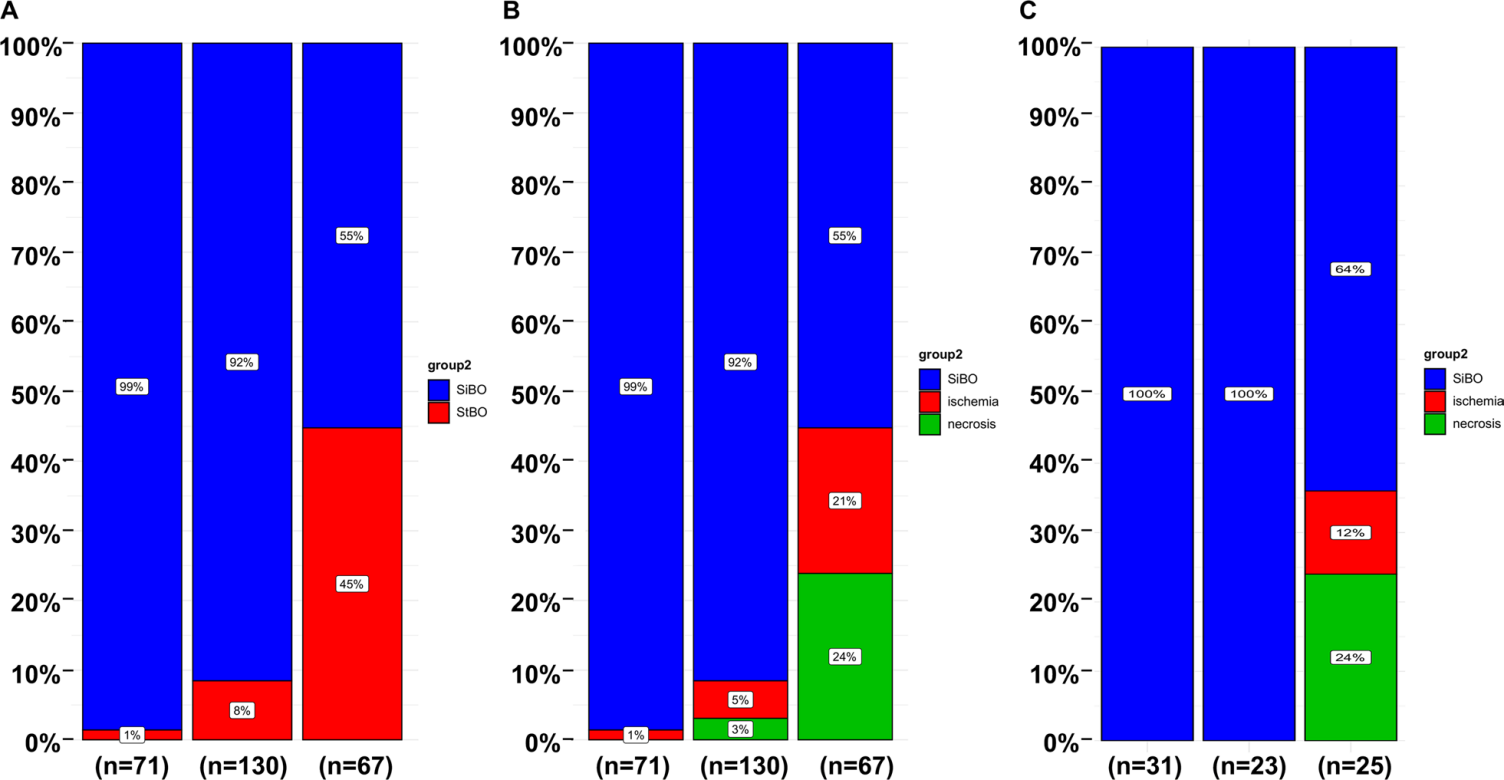
Validation of multi-dimensional models and external data for prediction of StBO in different status

Patients were further divided into three groups based on the fourth quartile of the multi-dimensional model scores: a low-risk group (risk scores ≤ − 3.091, n = 71), a medium risk group (− 3.091 < risk scores ≤ − 1.472, n = 130) and a high-risk group (risk scores > − 1.472, n = 67). Obviously, strangulated bowel was rarely observed in patients in the low-risk group (1%), but it was strongly associated with patients in the high-risk group (45%) (Fig. 5). The predictive value for the two cutoff points was as follows: a sensitivity of 97.6% and specificity of 40.0% for a lower score (− 3.091) and a sensitivity of 71.4% and specificity of 83.6% for a score of − 1.472. Moreover, to evaluate the properties of the model for predicting the degree of ischemia, we stratified the patients into a simple bowel ischemia group and a transmural necrosis group. The proportion of patients with transmural necrosis was significantly greater in the high-risk group (24%) than in the medium-risk group (3%). No transmural necrosis was found in the low-risk group (Fig. 6A and B).

In the validation set, The AUC of this multi-dimensional models was 0.910 (95%CI, 0.843–0.976) (Fig. 4.B). None of patients in the low-risk and medium-risk score group suffered with StBO. However, all patients with bowel ischemia (12%) and necrosis (24%) were resorted into high-risk score group (Fig. 6C).

## Discussion

SBO is always a dilemma for emergency surgeons in providing care. First, delayed surgery for StBO leads to severe complications such as intestinal ischemia, necrosis, perforation, peritonitis, sepsis, and multiple organ failure, with a dramatically increased mortality of 20–40%[34]. However, unnecessary surgery for SiBO may aggravate the formation of adhesive bands with subsequent adherence and its potential sequelea[29]. The prompt and accurate diagnosis of StBO still poses challenges for clinicians.

Previous studies have confirmed the discriminative efficacy of CT findings in the diagnosis of StBO, especially the presence of mesenteric fluid, ascites, edema of the bowel wall and whirl signs in CTA[12, 13, 21, 35]. Similarly, in our radiological analysis, mesenteric fluid, ascites, bowel spiral signs and edema of the bowel wall in emergency CT scans seemed closely related to StBO. Based on multivariate analysis, only ascites and bowel spiral signs were independent risk factors for StBO. This might be due to the classical characteristics of high metabolic activity and terminal artery perfusion in the small intestine mucosa. In the presence of mechanical SBO with mesenteric spirals, the permeability of the impaired mucosa increases[29, 33], which results in the transudative loss of fluid from the lumen into the peritoneal cavity. According to our etiology analysis, volvulus and hernias occupied a greater proportion of factors in StBO than in SiBO (Additional file 1: Table S1). Moreover, with the stasis of intestinal contents and bowel dilation, it may evolve into low or absent bowel sounds when SBO is aggravated, which could account for low-to-absent bowel sounds as an independent risk factor for StBO. The AUC of the radiological model based on emergency CT scans in our study reached only 0.706. In addition, CTA has been recommended as the gold standard for the diagnosis of bowel ischemia, with AUCs ranging from 0.87 to 0.91[8, 36]. The limitations include the potential risk of nephropathy induced by iodine, high costs and unavailability for most primary medical institutions[17], which hamper the performance of CTA.

**Fig. 7:**
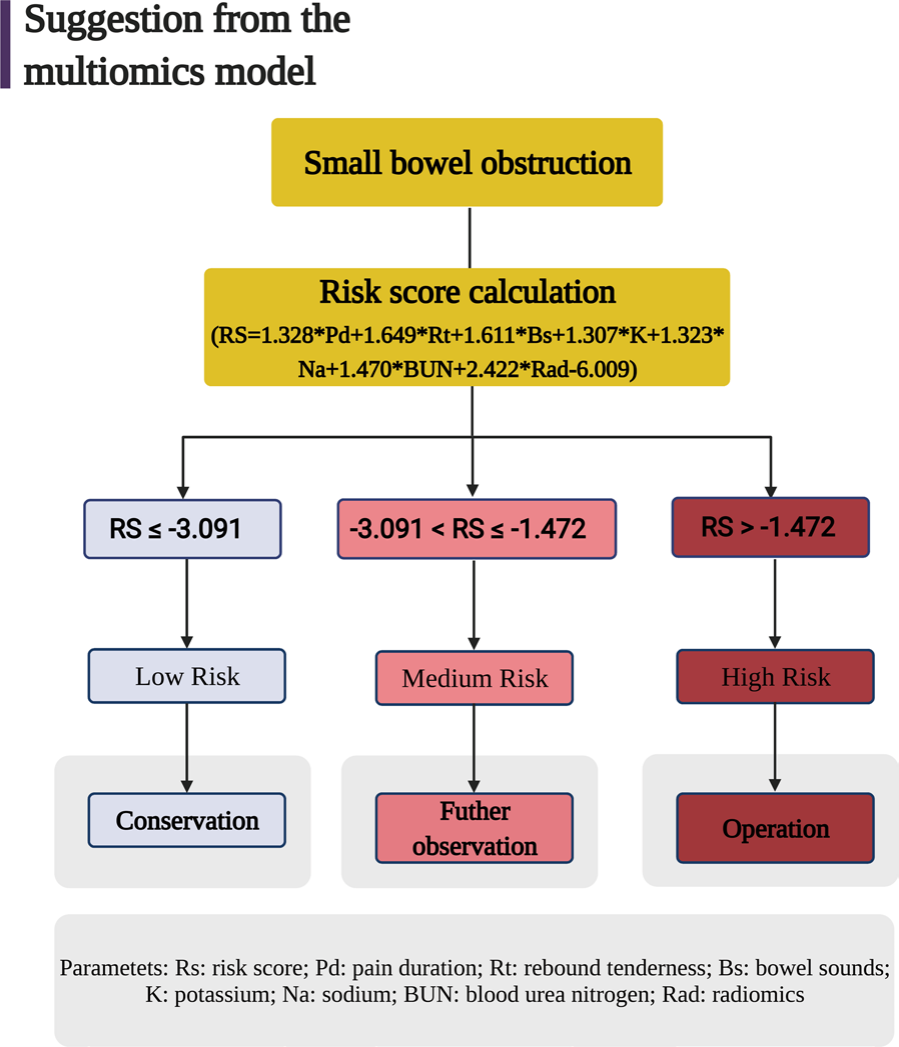
Proposal management for different calculated scores deriving from patient information. Rs: risk score; Pd: pain duration; Rt: rebound tenderness; Bs: bowel sounds; K: potassium; Na: sodium; BUN: blood urea nitrogen; Rad: radiological characteristics

Furthermore, we developed a multi-dimensional model based on clinical features, laboratory tests and radiological characteristics for the prediction of StBO. Once strangulated bowel develops, with increasing translocation of bacterial products from the intestinal lumen to blood circulation, a severe inflammatory response, including leukocytosis and neutrophilia, tends to occur[33, 37]. Similar to our findings, the levels of WBC and NE% were much higher in the StBO group, and the symptom of peritonitis with rebound tenderness was confirmed as an independent risk factor for StBO. An imbalance between the absorption and secretion of impaired intestinal mucosa also triggers electrolyte disturbances[2, 38]. In our multi-dimensional model, we defined hyponatremia, hypokalemia and rising levels of BUN as independent risk factors for StBO. In addition, insufficient renal perfusion due to extrasecretion in the intestinal lumen and the accumulation of lactic acid produced by intestinal anaerobic glycolysis deteriorate renal function with increasing levels of BUN in peripheral blood[22, 39]. Consequently, the distal convoluted tubule response to aldosterone results in the reabsorption of Na + by exchanging K + or H + , thus, hyponatremia and hypokalemia occur[2]. Usually, unlike a long pain duration indicating a chronic and reversible phase of disease, a short pain duration might indicate a status of acute and severe inflammation. Comprehensively, we constructed a multi-dimensional model for the prediction of StBO based on seven risk factors, including a radiological score, pain duration, bowel sounds, rebound tenderness, and the levels of sodium, potassium and BUN. The AUC of this multi-dimensional model was 0.857 (95% CI: 0.793–0.920), which was much higher than that of the model that only consisted of radiological characteristics[15] and equal to that of the previous CTA model[8, 36] (Additional file 1: Table S5). According to a previous study, we calculated the scores of our multi-dimensional model by summing the respective regression coefficients of the risk factors[22]. The formula is shown in Fig. 7. Furthermore, a nomogram was also constructed to reveal the weights for each factor, and radiological characteristics played a dominant role in predicting StBO. Secondary to radiological characteristics, the clinical symptoms were found to be crucial factors in the prediction of StBO.

Recently, most studies have focused on the prediction of bowel transmural necrosis in AMI[22, 23, 40], and few studies have focused on StBO. Although great advancements have been made in the detection of novel biomarkers associated with bowel ischemia[41–45], only I-FABP and PCT have been focused on in the prediction of bowel transmural necrosis with unsatisfactory accuracy[44, 45]. Here, by stratifying all patients into low-risk, medium-risk and high-risk groups according to their multi-dimensional scores (Fig. 6), we revaluated the discriminative ability of the multi-dimensional model for the prediction of transmural necrotic bowel obstruction. Excitedly, our models showed great efficacy not only for identifying patients with StBO but also recognizing transmural necrosis. Patients with bowel ischemia were primarily observed in the high-risk group, and the proportion of patients with bowel transmural necrosis was significantly higher than that in the medium-risk group. No transmural necrosis cases were found in the low-risk group. Only one patient in the low-risk group developed bowel ischemia without necrosis (Additional file 1: Table S4), which proved mild ischemia in this case. Additionally, another four patients with bowel transmural necrosis were observed in the medium-risk group. Although the specificity of our model largely improved with the rising score endpoint, there inevitably existed a loss of sensitivity, which is a shortcoming of the model. However, aggressive exploration is of greater importance than passively waiting for patients to show signs of suspected bowel transmural necrosis. Constant and dynamic observation is also necessary for patients in the low- or medium-risk group. This predictive model also well performed in our validation set.

The limitations of the present study are as follows. First, this study was a retrospective study conducted in a single center. Second, some parameters may not be identified due to the small-scale sample. Moreover, the validation for the multi-dimensional model is an internal validation only. Further efforts are needed in large-scale and prospective studies and effective external validations.

## Conclusion

The novel multi-dimensional model consisting of risk factors for pain duration, rebound tenderness, bowel sounds, potassium, sodium, and BUN levels and radiological characteristics offers a useful tool for predicting StBO. Clinical management can be performed according to the multi-dimensional score; for patients with low risk (scores ≤ − 3.91), conservative treatment is recommended. For the high-risk group (risk scores > − 1.472), there was a strong suggestion for detection with laparotomy. For the remaining patients (− 3.091 < risk scores ≤ − 1.472), dynamic observation is suggested.

## Supplementary Information

**Additional file1**: **Table S1.** The etiology of small bowel obstruction. **Table S2.** The definitions for CT findings. **Table S3.** The discriminative effectiveness of CT score. **Table S4.** Analyzation of the negative predicting results. **Table S5.** Previous studies on predictive model. **Table S6.** Treatments and outcomes of all patients. **Table S7.** Comparison of the clinical and radiological characteristics of the patients in external validation set.

## Data Availability

The datasets supporting the conclusions of this study are included within the article. Corresponding to Junrong Zhang and Xianqiang Chen when necessary.

## Abbreviations

StBO: Strangulated bowel obstruction
SiBO: Simple bowel obstruction
ROC: Receiver operating characteristic
AUC: Area under the curve
AMI: Acute mesentery ischemia
BMI: Body mass index
WBC: White blood cell
NE%: Neutrophil percentage
PCT: Procalcitonin
BUN: Blood urea nitrogen
PT: Prothrombin time
DDI: D-dimer
CTA: Computed tomography angiography
