# Supplementary material for: Prediction and management of strangulated bowel obstruction: a multi-dimensional model analysis
Source: BMC Gastroenterol. 2022 Jun 22;22:304. doi: 10.1186/s12876-022-02363-1 (PMC9219133; doi:10.1186/s12876-022-02363-1)
Supplement: Supplementary file 1 — Additional file1: Table S1. The etiology of small bowel obstruction. Table S2. The definitions for CT findings. Table S3. The discriminative effectiveness of CT score. Table S4. Analyzation of the negative predicting results. Table S5. Previous studies on predictive model. Table S6. Treatments and outcomes of all patients. Table S7. Comparison of the clinical and radiological characteristics of the patients in external validation set. [file 12876_2022_2363_MOESM1_ESM.docx]

**Supplementary table 1:The etiology of small bowel obstruction.**

| Etiology | SiBO (n=236) | StBO (n=45) | *p*-value |
| --- | --- | --- | --- |
|  | | | **<0.001** |
| Adhesion | 147(62.3%) | 18(40.0%) |  |
| Volvulus | 16(6.8%) | 11(24.4%) |  |
| Intussusception | 6(2.5%) | 1(2.2%) |  |
| Intestinal bezoar | 13(5.5%) | 2(4.4%) |  |
| Tumor | 15(6.4%) | 0 |  |
| Inflammatory bowel disease | 9(3.7%) | 0 |  |
| Anastomotic stenosis | 1(0.4%) | 0 |  |
| Hernia | 8(3.4%) | 10(22.1%) |  |
| Ischemic bowel disease | 2(0.8%) | 3(6.6%) |  |
| Other diseases | 13(5.5%) | 0 |  |
| NA | 6(2.5%) | 0 |  |

NA:not accessible

**Supplementary table 2:The definitions for CT findings.**

| **CT characteristics** | **Definition** | **Quotation** |
| --- | --- | --- |
| Mesenteric fluid | hazy mesentery of the involved intestinal segments | [23] |
| Ascites | free fluid accumulated in physical pouch or between-loops | [24] |
| Spiral signs | mesenteric vessels and bowel loops twisted together | [24, 25] |
| Concentric circle signs | pathognomonic bowel-within bowel configuration, appears as a sausage-shaped mass when CT images is obtained parallel to its longitudinal axis of digestive tract but as a target-like mass when CT images is perpendicular to the cross sections of digestive tract | [26] |
| Small bowel feces signs | the presence of mottled, feculent material, resembling colonic contents, in dilated small bowel immediately proximal to the transition point | [4, 27] |
| Edema of bowel wall | bowel wall greater than 3mm in thickness | [4] |

**Supplementary table 3: the discriminative effectiveness of CT score.**

|  | SiBO(n=236) | StBO(n=45) | *p*-value |
| --- | --- | --- | --- |
| score |  | | **＜0.001** |
| 0,n(%) | 71(93.4%) | 5(6.6%) |  |
| 1,n(%) | 150(87.2%) | 22(12.8%) |  |
| 2,n(%) | 15(45.5%) | 18(54.5%) |  |

**Supplementary table 4: Analyzation of the negative predicting results.**

|  | Pd | Bs | Rt | K+ | Na+ | BUN | Ascites | Spiral signs |
| --- | --- | --- | --- | --- | --- | --- | --- | --- |
| Patient1* | - | + | - | + | - | - | + | - |
| Patient2 | + | + | - | - | + | - | + | - |
| Patient3 | + | + | - | - | - | + | + | - |
| Patient4 | - | + | + | - | - | - | + | - |
| Patient5 | + | + | - | - | - | + | + | - |

Pd: pain duration; Rt: rebound tenderness; Bs: bowel sound; K: potassium; Na: sodium; BUN: blood urea nitrogen; Patient1*: an entity from low risk group with strangulated bowel. Patient2-5: patients from medium risk group with necrosis bowel.

**Supplementary table 5: Previous studies on predictive model.**

| **Year** | **Sample Size(n)** | **Strangulated cases (n)** | **Risk Factors** | **Auc(95%CI)**  **/C-index** |
| --- | --- | --- | --- | --- |
| 2010 | 100 | 11 | Vomiting, small bowel feces sign, free intraperitoneal fluid, mesenteric edema | C-index =0.75 |
| 2010 | 233 | 45 | leucocyte, C-reactive protein, ascites, reduced wall contrast enhancement | 0.88 (0.80–0.97) |
| 2010 | 154 | 25 | age, presence of ascites, drainage volume | 0.88(0.80–0.94) |
| 2015 | 202 | 52 | no flatus, free fluid, high-grade obstruction | 0.75 (0.66-0.88) |
| 2017 | 417 | 76 | temperature, peritoneal sign, WBC count, ascites, thick-walled small bowel≥3mm | 0.935(0.900-0.969) |
| 2020 | 124 | 43 | age，pain duration before admission，body temperature, WBC, reduced wall enhancement, segmental mesenteric fluid | 0.92 (0.85–0.98) |
| 2021 | 236 | 45 | pain duration, rebound tenderness, none or low or normal bowel sound, potassium level, sodium, BUN, Ascites, bowel spiral signs | 0.857(0.793–0.920) |

**Supplementary table 6: Treatments and outcomes of all patients.**

|  | SiBO (n=236) | StBO (n=45) | *p*-value |
| --- | --- | --- | --- |
| Management, n(%) |  |  | **<0.001** |
| surgery | 57(24.2%) | 43(95.6%) |  |
| conservative treatment | 179(75.8%) | 2(4.4%) |  |
| Severe adverse event, n(%) |  |  | **<0.001** |
| none | 228(98.7%) | 37(82.2%) |  |
| yes | 3(1.3%) | 8(17.8%) |  |
| ICU management, n(%) |  |  | **<0.001** |
| none | 228(98.7%) | 37(82.2%) |  |
| yes | 3(1.3%) | 8(17.8%) |  |
| Length of stay(medium) | 7 | 16 | **<0.001** |
| Fees(¥, medium) | 16791.00 | 59563.00 | **<0.001** |

SiBO: simple bowel obstruction; StBO: strangulated bowel obstruction;

ICU management: intensive care unit management.

**Supplementary table 7: Comparison of the clinical and radiological characteristics of the patients in external validation set.**

| Characteristics | SiBO(n=71) | StBO(n=9) | *p*-value |
| --- | --- | --- | --- |
| Gender, n(%) |  |  | 0.712** |
| male | 47(66.2%) | 5(55.6%) |  |
| female | 24(33.8%) | 4(44.4%) |  |
| Age(median) | 60 | 64 | 0.389***** |
| BMI, n(%) |  |  | 0.153** |
| 18.5-23.9 | 37(64.9%) | 3(33.3%) |  |
| ≤18.5 | 13(22.8%) | 4(44.4%) |  |
| ＞23.9 | 7(12.3%) | 2(22.2%) |  |
| Glucose(median) | 7.15 | 8.99 | 0.354 |
| BUN, n(%) |  |  | 1.000** |
| <8.3 | 55(78.6%) | 7(77.8%) |  |
| >8.3 | 15(21.4%) | 2(22.2%) |  |
| Comorbidity, n(%) |  |  | 0.865** |
| none | 57(80.3%) | 8(88.9%) |  |
| yes | 14(19.7%) | 1(11.1%) |  |
| Pain duration, n(%) |  |  | 0.209** |
| ≤3days | 35(49.3%) | 7(77.8%) |  |
| ＞3days | 36(50.7%) | 2(22.2%) |  |
| History of abdominal operation, n(%) |  |  | 0.205** |
| none | 15(21.1%) | 4(44.4%) |  |
| yes | 56(78.9%) | 5(55.6%) |  |
| Temperature(median) | 37.2 | 37.1 | 0.516* |
| Rebound tenderness, n(%) |  |  | **<0.001**** |
| yes | 7(9.9%) | 6(67.6%) |  |
| WBC, (10^9/L), n(%) |  |  | **0.039**** |
| ≤75% quartile | 56(78.9%) | 4(44.4%) |  |
| ＞75% quartile | 15(21.1%) | 5(55.6%) |  |
| NE%, n(%) |  |  | **0.006**** |
| ≤75% quartile | 57(80.3%) | 3(33.3%) |  |
| ＞75% quartile | 14(19.7%) | 6(66.7%) |  |
| Ascites |  |  | **0.003**** |
| yes | 34(47.9%) | 9(100%) |  |
| Spiral signs |  |  | 0.080** |
| yes | 7(9.9%) | 3(33.3%) |  |

SiBO: simple bowel obstruction; StBO: strangulated bowel obstruction;

Values marked with “*” were compared using Wilcoxon rank-sum test.

Values marked with “**” were adjusted *p*-values.

**Supplementary table 8: Analyzation of correlation between the features in multi-dimensional model.**

| Phi/Cramer’s V | Pd | Rt | Bs | K | | Na | BUN | Rad |
| --- | --- | --- | --- | --- | --- | --- | --- | --- |
| Pd |  | 0.035 | 0.007 | | 0.225 | 0.095 | 0.041 | 0.040 |
| Rt |  |  | 0.036 | | 0.051 | 0.036 | 0.003 | 0.131 |
| Bs |  |  |  | | <0.001 | 0.027 | 0.028 | 0.061 |
| K |  |  |  | |  | 0.111 | 0.063 | 0.020 |
| Na |  |  |  | |  |  | 0.009 | 0.098 |
| BUN |  |  |  | |  |  |  | 0.136 |

Rs: risk score; Pd: pain duration; Rt: rebound tenderness; Bs: bowel sound; K: potassium; Na: sodium; BUN: blood urea nitrogen; Rad: radiological score
